# Toward Precision Medicine in Celiac Disease: Emerging Roles of Multiomics and Artificial Intelligence

**DOI:** 10.1155/ancp/6570955

**Published:** 2025-10-03

**Authors:** Moein Piroozkhah, Mozhgan Foroutan Kahangi, Nastaran Asri, Mobin Piroozkhah, Niloofar Moradi, Ehsan Nazemalhosseini-Mojarad, Zahra Salehi, Mohammad Rostami-Nejad

**Affiliations:** ^1^Basic and Molecular Epidemiology of Gastrointestinal Disorders Research Center, Research Institute for Gastroenterology and Liver Diseases, Shahid Beheshti University of Medical Sciences, Tehran, Iran; ^2^Gastroenterology and Liver Diseases Research Center, Research Institute for Gastroenterology and Liver Diseases, Shahid Beheshti University of Medical Sciences, Tehran, Iran; ^3^School of Medicine, Tehran University of Medical Sciences, Tehran, Iran; ^4^Cell Therapy and Hematopoietic Stem Cell Transplantation Research Center, Research Institute for Oncology, Hematology and Cell Therapy, Tehran University of Medical Sciences, Tehran, Iran; ^5^Hematology, Oncology and Stem Cell Transplantation Research Center, Research Institute for Oncology, Hematology and Cell Therapy, Tehran University of Medical Sciences, Tehran, Iran; ^6^Celiac Disease and Gluten-Related Disorders Research Center, Research Institute for Gastroenterology and Liver Diseases, Shahid Beheshti University of Medical Sciences, Tehran, Iran

**Keywords:** artificial intelligence, CD, diagnosis, gluten-related disorders, machine learning, multiomics, precision medicine

## Abstract

This paper focuses on celiac disease (CD), an autoimmune disorder characterized by small intestinal enteropathy and gastrointestinal symptoms. The prevalence of CD is rising, and there is a need for prompt and accurate diagnosis. The paper discusses the challenges in classifying CD and the consequences of delayed diagnosis, including increased morbidity and mortality rates. It also highlights the Marsh classification, which categorizes CD based on histological characteristics. However, this classification has limitations, which can lead to misdiagnosis. The emergence of multiomics data and artificial intelligence (AI) is presented as a potential solution to improve CD diagnosis and management. Multiomics data integration enables a more precise diagnosis, while AI and machine learning (ML) techniques facilitate the identification of clinically relevant patterns and the development of diagnostic models. According to the findings, the integration of AI and multiomics data holds transformative potential for the diagnosis and management of CD, facilitating early diagnosis and personalized treatment strategies. AI-assisted methods, including video capsule endoscopy (VCE) analysis, have shown sensitivities exceeding 90%, enhancing diagnostic accuracy while reducing invasiveness. As novel biomarkers are identified, individualized management approaches can evolve, leading to improved patient outcomes, though further efforts are needed to standardize these technologies in clinical practice.

## 1. Introduction

Celiac disease (CD), a type of autoimmune disorder, is characterized by small intestinal enteropathy, immunological activation, gastrointestinal symptoms related to malabsorption, and the presence of autoantibodies to tissue transglutaminase (tTG). As CD is more understood and detected through testing, its prevalence has been rising recently. Additionally, despite disease identification, increased prevalence is due to an actual rise in this immune-based disease [1]. Initially, CD was thought to be a condition that only affected children. Still, it has recently been discovered that this condition frequently appears in adulthood, even in the seventh and eighth decades of life [2]. The incidence of CD is estimated to be between 0.5% and 1% in the general population, making it one of the most prevalent autoimmune diseases [3]. The prevalence rate is 1.4% according to results from serologic tests and 0.7% based on information obtained from biopsy [4]. This finding demonstrates that the diagnostic evaluation via the biopsy test is correct in only approximately half of the cases, compared to those obtained with serologic tests. Gastroduodenoscopy is the gold standard for CD diagnosis and the most citable way for detecting CD, and this highlights that celiac misdiagnosis is a severe issue.

Recent advancements in artificial intelligence (AI), a field that encompasses machine learning (ML) and deep learning techniques, have revolutionized medical diagnostics [5, 6]. AI-powered tools, such as ML and deep learning models, are increasingly being applied to analyze complex datasets, enhance diagnostic accuracy, and predict disease progression. Nowadays, AI has a promising effect on clinicians' workflow, accurate diagnosis, and disease management for patients [7]. For instance, AI has demonstrated remarkable success in medical imaging and diagnostics, enabling early detection of conditions like cancer and neurological diseases through pattern recognition in radiological and histological data [8]. Notably, in thyroid cytopathology, quantitative nuclear analysis using AI has successfully distinguished between papillary thyroid carcinoma and benign nodules with high accuracy by objectively measuring subtle morphological features (nuclear area and elongation) that are challenging for human observers [9]. These techniques show particular promise for resolving indeterminate thyroid nodule classifications, where AI has demonstrated improved accuracy and standardization of cytological diagnosis [10]. These technologies could address current challenges in CD diagnosis, such as reducing reliance on invasive procedures and improving the interpretation of serologic and biopsy results.

Omics refers to a comprehensive and systematic approach that encompasses various biological layers, including genomics (the study of an organism's complete set of DNA), transcriptomics (the analysis of RNA transcripts), proteomics (the identification and quantification of proteins), metabolomics (the study of metabolic profiles), and microbiomics (the exploration of microbial communities) [11]. By integrating these diverse omics data, researchers can obtain a holistic view of biological processes and disease mechanisms, enabling the identification of potential biomarkers and therapeutic targets [12].

Precision psychiatry, pharmacology, tumor grading, and management, such as in the fields of lung, breast, and glioma, are some areas where AI and multiomics data have made significant impacts [13–16]. The integration of AI and multiomics data has become a significant strategy in precision medicine. A ML model analyzing multiomics data from pancreatic ductal adenocarcinoma patients revealed a high accuracy rate in disease survival prediction [17]. Additionally, various ML and deep learning models utilized multiomics data to predict leukemia with an accuracy rate of over 95%, indicating the high potential of these novel models in patient management and care [18]. Recently, researchers have utilized new ML and deep learning models to study CD, which are trained on multiomics data. The results demonstrate a hopeful future for novel management and treatment options [19, 20].

**Figure 1:**
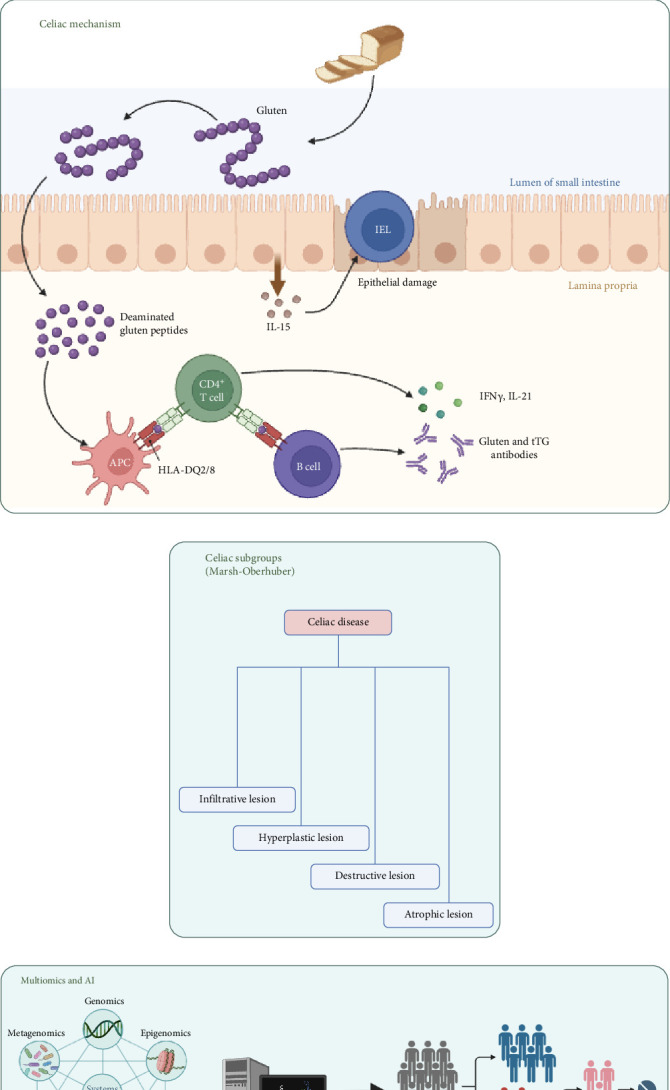
Schematic representation of key concepts covered in the review. (A) Disease overview and epidemiology, (B) molecular pathogenesis, (C) histological classification, and (D) recent advances in multi-omics and artificial intelligence.

Therefore, this review aims to elucidate the challenges inherent in current CD diagnostic practices, including classification difficulties and the consequences of delayed diagnosis, while highlighting the transformative potential of innovative methodologies. By emphasizing the integration of multiomics and AI technologies, we aim to pave the way for personalized medicine approaches that can significantly improve patient outcomes in CD management (Figure 1).

## 2. Methodology

We conducted a literature review using databases such as PubMed, Scopus, and Google Scholar to identify relevant studies published up to December 2023. Keywords included “celiac disease,” “multi-omics,” “artificial intelligence,” “machine learning,” and “gluten-related disorders.” The inclusion criteria encompassed peer-reviewed articles, clinical trials, and meta-analyses that focused on integrating multiomics data with AI technologies to enhance diagnostic accuracy and treatment outcomes in CD. Additional articles were identified by searching the reference lists of the included studies.

## 3. Results

### 3.1. The Classification Obstacles in CD

**Figure 2:**
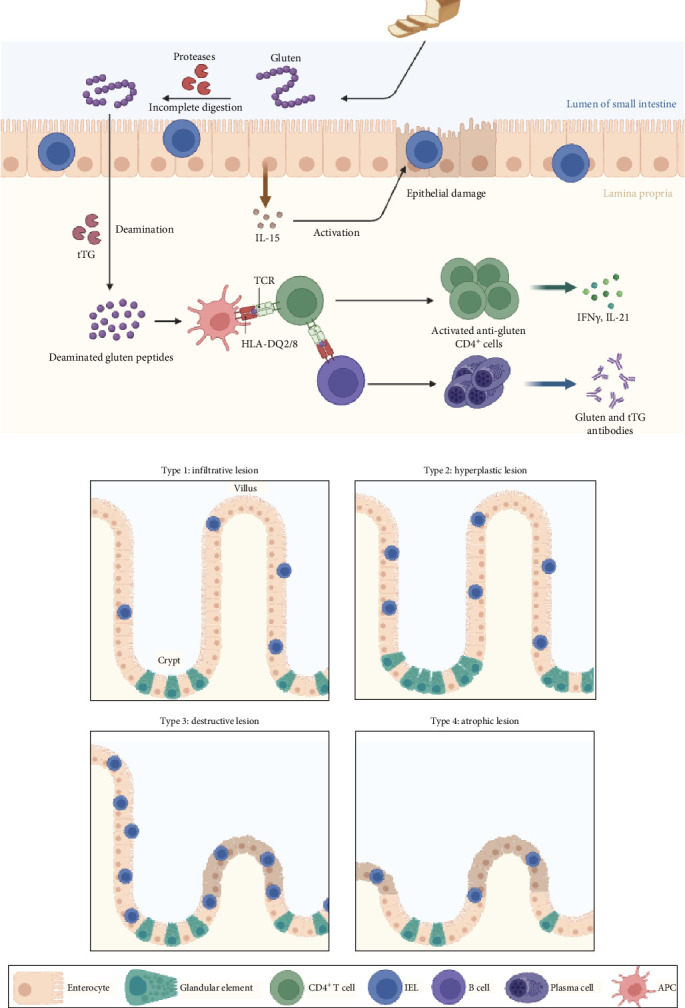
Different aspects of CD pathology. (A) The Immune Response to gluten in CD. It begins with the incomplete digestion of gluten in the lumen of the small intestine. Gluten peptides then interact with tissue transglutaminase (tTG), which deaminates them, increasing their affinity for HLA-DQ2/8 on antigen-presenting cells (APCs). This interaction occurs in the lamina propria, a layer of connective tissue under the intestinal epithelium. The activated APCs present the deaminated gluten peptides to CD4^+^ T cells via the T-cell receptor (TCR), activating these T cells. Subsequently, the activated CD4^+^ T cells proliferate and produce proinflammatory cytokines, such as IFN-γ and IL-21. These cytokines contribute to the epithelial damage observed in CD and the production of autoantibodies against gluten and tTG. (B) The progressive stages of intestinal damage in CD. The histological changes in the small intestine associated with CD are classified into four types of lesions. Type 1 is an infiltrative lesion characterized by increased intraepithelial lymphocytes (IELs) but with normal villous architecture. Type 2, the hyperplastic lesion, shows increased IELs and crypt hyperplasia. Type 3, the destructive lesion, exhibits a more pronounced increase in IELs, crypt hyperplasia, and villous atrophy. Lastly, Type 4, the atrophic lesion, is marked by total villous atrophy and crypt hyperplasia. The different cell types involved in the immune response, such as enterocytes, glandular elements, CD4^+^ T cells, IELs, B cells, plasma cells, and APCs, are color-coded and listed at the bottom for reference.

From a single perspective, CD is a subgroup of gluten-related diseases (GRDs) that encompasses CD, wheat allergy, and non-celiac gluten sensitivity (NCGS). With a predicted global incidence of nearly 5%, these subgroups have emerged as a significant epidemiological phenomenon. Despite the fact that they share comparable clinical signs and specific pathogenetic mechanisms that contribute to their development, it might be difficult to differentiate between them [21]. In celiac, gluten-derived peptides cause a T-cell mediated autoimmune response, and this event occurs in the small bowel and results in the characteristic enteropathy and malabsorption syndrome (Figure 2A) [22].

HLA-DQ2 heterodimer is expressed by nearly all (95%) of CD patients, and HLA-DQ8 is present in the majority of the remaining cases [23]. Indeed, in 95% of cases, the diagnosis of CD can be ruled out by their absence. Besides this, the HLA DQ2 and/or DQ8 genes may be present in up to 50% of NCGS patients. Additionally, approximately 30% of healthy individuals exhibit these types of HLAs; therefore, these genes cannot be used as a distinguishing characteristic between different types of GRDs [24]. In the absence of CD or WA, patients with NCGS typically report a range of intestinal and extraintestinal symptoms that appear soon after consuming gluten-containing foods. The current belief is that there is a nonautoimmune, nonallergic process, even though the pathogenetic mechanisms triggering the beginning of NGCS are still far from being fully understood [25]. In patients with CD, the consumption of gluten can trigger a T-cell-mediated immune response against tTG, an extracellular matrix enzyme, which can damage the mucosa and ultimately lead to intestinal villous atrophy [21]. Researchers are still looking at the histologic features of NCGS, which include reports of what appears to be regular histology and a modest increase in T cells in the superficial epithelium in normal villi [24]. Opposed to CD, the diagnosis of NCGS still faces a significant problem due to the absence of defined biomarkers; clinical symptoms are vague and could be mistaken for those of other illnesses, such as IBS [26]. Recent studies about NCGS' pathogenesis indicate that there is a need to find more effective ways of managing gluten sensitivity-related diseases. The gluten-free diet has almost no effect on patients' symptoms, which means the diagnosis and management of NCGS should be entrusted to new methods [27]. Along with these difficulties, it can be quite challenging for pathologists to make an accurate and timely diagnosis of CD categorization. An early CD diagnosis is crucial to lowering the risk of cancer, preventing cumulative morbidity and premature death, in addition to improving the quality of life for patients [28].

### 3.2. Delayed Diagnosis and Its Consequences

The majority of research demonstrates a slight increase in morbidity and mortality rates following a clinical CD diagnosis. Patients with CD have higher standardized mortality ratios, based on both clinical and histological evidence [29]. There was a 2.8-fold higher risk of death in people with type I diabetes with a concomitant diagnosis of CD compared to those who had the diagnosis for more than 15 years without CD [30]. Additionally, we can mention the benefit of gluten-free diets for autoimmune thyroid disease accompanied by CD; this means that a delayed diagnosis of CD may cause delayed benefit of the gluten-free diet [31]. There are statistical analyses that demonstrate the prevalence of undiagnosed CD, such as those from the US cohort research [13], where, surprisingly, 83% of the cases in this group of people with positive serology had not been previously diagnosed with CD. According to these figures, the vast majority of CD patients in the US remain undiagnosed. In addition to this, a recent study from Sweden found that the median duration from the onset of symptoms to diagnosis was 9.7 years, and the median time from the patient's first visit to the doctor to diagnosis was 5.8 years [32]. Some authors have hypothesized that the long-term dietary consumption of gluten is a risk factor for developing cancer [33]. This risk is more significant for gastrointestinal cancers, such as non-Hodgkin lymphoma and small bowel carcinoma [34]. Moreover, patients with CD who remain untreated are more likely to develop lymphoma [35]. In combination, these results underscore the importance of early diagnosis for enhanced patient management in CD. A precise puzzle-like reconstruction of the clinical, serological, genetic, and histological aspects is necessary for a proper diagnosis of CD [36].

### 3.3. Marsh Classification

Based on histological characteristics, CD is split into different diagnostic groups using the Marsh classification. Following Marsh's initial definition, the spectrum of mucosal pathology in CD has undergone several revisions by Oberhuber, Corazza, Villanaci, and eventually Ensari [37]. The most popular approach is the Marsh-Oberhuber classification; however, no method is used universally [38]. The Marsh-Oberhuber classification outlines five distinct types of small-intestinal mucosal injury associated with CD: Type 0 is characterized by normal mucosa with fewer than 30 intraepithelial lymphocytes (IELs) per 100 enterocytes. Type 1 features normal villous architecture with increased IELs (≥30 per 100 enterocytes). Type 2 combines infiltrative changes with crypt hyperplasia, also maintaining normal villous architecture. Type 3 is the destructive type, subcategorized into three groups based on the severity of villous atrophy: Type 3a displays mild atrophy, Type 3b shows marked atrophy, and Type 3c indicates total atrophy. Finally, Type 4 represents a rare atrophic pattern with only a few crypts and near-normal IEL counts, typically associated with more severe conditions like refractory sprue or enteropathy-associated T cell lymphoma (Figure 2B) [36, 39]. Similar to other categorization, this one has issues such as the failure to adequately explain the condition of the majority of patients, who frequently come with milder lesions lacking of “atrophy.” In fact, pathologists may easily ignore small mucosal abnormalities, and when they are discovered, they may be connected to various disorders in addition to CD [37]. Since the diagnosis of the Marsh classification depends on the pathologists' final decision, and they may have differing perspectives on the same results, the possibility of misdiagnosis is extremely high. For instance, a study revealed that different pathologists within the same department had more agreement about biopsy evaluation than other pathologists from separate hospitals. These results highlight the impact of local procedures on the accuracy of the pathological diagnosis and also show that pathologists may arrive at different conclusions based on the same information across different centers. Therefore, it can be extremely useful to apply a method that minimizes human error or interpretation differences by also using today's knowledge of multiomics data and AI.

### 3.4. The Emergence of Multiomics and AI

The data sets coming from several omics analyses can be grouped during the analysis step and analyzed jointly. This concept is generally referred to as “multiomics.” Genomics, transcriptomics, metabolomics, proteomics, microbiomics, and radiomics are the most commonly applied omics groups in multiomics studies. Each group of omics data often provides a list of categories related to the different conditions. These findings can be used to identify illness-related biological pathways or processes as well as to shed light on how the disease and control groups differ from each other [40, 41]. These groups of omics can provide a better understanding of the image signals of the tumor microenvironment, [42] the function of genes, and their relationship with various diseases [43], as well as metabolic pathways that can be manipulated for health benefits [44]. According to research findings, the development of CD requires both a genetic susceptibility and the effects of gluten consumption, but neither alone is sufficient to induce CD [45]. This suggests that exposure to other environmental factors may also be necessary for the development of CD, which can alter gene expression and subsequently protein levels (through transcription and translation effects). Multiomics provides a platform to consider all these factors simultaneously.

In addition to this multiomics approach, which can provide more focused data for a more precise diagnosis, AI, a general term that denotes the use of computers to model intelligent behavior, can minimize the need for human intervention [5]. AI affects three levels of medicine. First of all, clinicians can have a rapid image interpretation and diagnosis. Then, patients can access all the data that they need to improve their health condition. Also, the health system's workflow improves [7]. AI and ML are both able to identify clinically relevant patterns, which will lead to the stratification of patients and provide a pathway for care, estimation of diseases, diagnosis, and management of illnesses. Due to the complex nature of data management in ML, it can be applied in biomedical research, precision medicine, and computer-aided diagnosis to enhance the global healthcare system [46].

The emergence of multiomics technologies has enabled researchers to access multilayered information about the genome, proteome, transcriptome, metabolome, and microbiome. These multidimensional data can be processed by AI in a health system, such as the cancer field, to develop precision medicine [12]. The role of ML in proteomics studies is becoming increasingly significant, which means an improvement in the quality and reliability of proteomics analysis [47]. Recent studies have demonstrated the advancement of radiomics analyses using AI, which leverages computed tomography (CT) scans, magnetic resonance imaging (MRI), and positron emission tomography (PET) scans. The integration of radiomics and AI can help radiologists predict a patient's future condition [48].

Hence, integrating AI and multiomics data analysis can enhance the clinician's ability to comprehend and manage diseases like celiac.

### 3.5. AI and Multiomics Trace in CD

**Figure 3:**
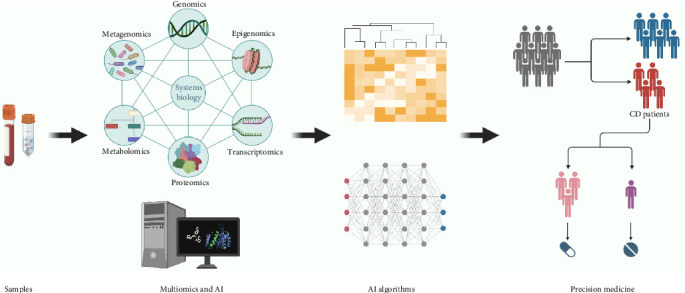
Artificial intelligence and multiomics in CD. The process of integrating multiomics data and artificial intelligence (AI) to advance precision medicine, particularly in the context of celica disease (CD) patients. Biological samples, such as blood, are collected. These samples undergo various types of “omics” analyses, which include genomics, epigenomics, transcriptomics, proteomics, and metabolomics. The interconnections among these omics-based techniques are depicted, emphasizing a systems biology approach. This multiomics data is then processed and analyzed using AI, shown as a computer performing complex analyses. The AI algorithms are visualized as a network of interconnected nodes, which represent the machine learning process. This may involve clustering or classifying patients based on the omics data. The output of the AI algorithms is used in precision medicine. A group of people is shown, with some individuals highlighted, representing the identification of CD patients from a larger population. This stratification leads to personalized treatment pathways, as indicated by the different pills and their corresponding matches to the patients.

AI has revolutionized the field of medical diagnostics, bringing significant changes to the ever-changing landscape. This technological advancement has had a particularly transformative effect on complex diseases, such as CD (Figure 3 and Table 1).

Traditionally, diagnosing CD has been challenging, requiring invasive upper digestive endoscopy and histological assessment of duodenal biopsies. These procedures not only cause discomfort and carry risks for patients, but they also add an additional task to the already laborious diagnostic process. However, the adoption of capsule endoscopy (CE) has led to a paradigm shift in the diagnostic journey for CD. By providing high-quality, magnified images of the small bowel mucosa, CE offers a noninvasive alternative to traditional methods. Several computer-assisted systems are utilizing neural convolutions to analyze images from endoscopes, aiming to improve the speed and accuracy of diagnosing CD. This approach has the potential to transform diagnostic processes, offering both time-saving and risk-reducing benefits. The development of these systems began in 2008 and has since seen over 50 publications exploring various techniques, such as spatial, transform, scale-invariant, and spatio-temporal methods [69]. However, the real game-changer emerged when AI, ML, and deep learning were integrated, tapping into the immense computing capabilities of graphics processing units to process the massive volume of digital images and medical records produced in the medical imaging field [70].

For example, the study by Ciaccio et al. [49] aimed to classify video CE (VCE) images to distinguish between CD patients and controls using quantitative image analysis. The AI models employed included a threshold classifier and an incremental learning classifier, both based on grayscale brightness and texture features extracted from 10 × 10-pixel subimages. The dataset consisted of 11 celiac patients and 10 controls, with 200 images per videoclip analyzed across five small intestinal regions. Validation was performed using a split of exemplar (training) and test sets, achieving sensitivities of 80%–88% and specificities of 80%–96%. Key limitations included the small sample size, exclusion of clips with excessive fluid/bubbles during training, and the manual selection of features and classifiers. Heterogeneity in CD presentation (e.g., patchy villous atrophy) may also affect generalizability [49].

Furthermore, the study of Wang et al. [50] proposed a novel block-wise channel squeeze and excitation (BCSE) module integrated into ResNet50 and Inception-v3 for CD diagnosis using VCE images. The dataset comprised 2140 images (1040 CD, 1100 controls) from 12 patients and 13 controls, which is relatively small and may limit generalizability. Validation used 10-time 10-fold cross-validation, demonstrating high accuracy (95.94%) but with potential overfitting risks due to limited patient-wise diversity [50].

Also, a group of researchers trained a ML model using the cases of biopsy-proven CD patients to measure the severity of CD. The MLA was trained on 334,080 frames from 35 CD patients and validated on 63 patients. The MLA showed strong agreement with experts (Krippendorff's α >0.8). Overall, the variability among experts in assessing CD severity, based on years of experience, highlights the need for a standardized diagnostic and monitoring ML model [51].

Another groundbreaking study has revealed an innovative approach for diagnosing CD. Stoleru and colleagues proposed a non-deep learning approach using modified Sobel and custom filters to detect CD from VCE images, focusing on texture analysis and artifact detection (e.g., mucosal atrophy and cracks). The dataset comprised 109 videos (45 healthy, 65 CD) from the PillCam SB3, split into 51 training, 51 test, and seven real-time validation videos. Validation used standard performance metrics, achieving 94.1% accuracy with linear SVM. What is even more remarkable is that this level of accuracy rivals that of more complex algorithms, proving that computer-assisted diagnosis of CD is possible without the need for costly equipment or lengthy processing [52].

In another investigation, a ResNet-50-based deep learning model was developed to classify CD from duodenal biopsy slides (gold standard). The dataset comprised 1230 whole-slide images (from 1048 patients), split into 1018 training images and 212 test images. The model achieved high accuracy (95.3% for CD) but was trained on single-center data (Dartmouth-Hitchcock Medical Center), risking bias. Limited samples for nonspecific duodenitis (64 slides) reduced performance for this class (F1:81%) [53].

The study by Koh et al. [54] aimed to automate the detection and classification of CD using duodenal biopsy images graded by Marsh scores. The researchers employed conventional ML techniques, including Random Forest, SVM, KNN, Adaboost, Bagged Trees, and Discriminant Subspace classifiers, combined with Steerable Pyramid Transform for feature extraction. The dataset consisted of 120 biopsy images (82 from CeD patients and 38 controls) across Marsh I-IIIC grades. Validation was performed using 10-fold cross-validation. The model achieved strong performance with 88.89% accuracy for H&E stained images, 82.92% for RGB images, and 72% for multiclass classification [54].

Additionally, Syed et al. [55] developed a convolutional neural network (CNN) to differentiate duodenal biopsies from children with environmental enteropathy (EE), CD, and healthy controls. The dataset included 3118 images from 102 patients, with 10-fold cross-validation used for model evaluation. The CNN achieved 93.4% case-detection accuracy and a false-negative rate of 2.4%, with most misclassifications occurring between CD and healthy biopsies. A deconvolutional neural network (DNN) was paired with the CNN to identify microlevel features, such as secretory cell alterations, aiding in disease differentiation [55].

The study by Gruver et al. [56] aimed to develop pathologist-trained ML classifiers to objectively quantify CD features (villus blunting, IELs, and crypt hyperplasia) in duodenal biopsies. The dataset size was limited (116 biopsies total, with only 10 used for training), and class imbalance was noted (e.g., few Type 2/3c cases). Validation relied on correlation with manual Marsh scores and paired pre- and post-diet biopsies, achieving 96.4% alignment in response assessment. However, the DenseNet2 CNN model's performance was influenced by biopsy orientation and sample quality, and the weakest correlation was observed for crypt hyperplasia (*p*=0.1369), mirroring known challenges in manual scoring [56].

Beyond imaging, advancements in AI are extending to serological or genetic diagnosis. For instance, Choung et al. [57], employed a fluorescent peptide microarray platform combined with ML models (random forest and support vector machines [SVM]) to identify immunogenic epitopes of the tTG-DGP complex for CD diagnosis and monitoring, using serum samples from 90 CD patients and 79 healthy controls in the discovery cohort and validating findings in 82 newly diagnosed CD patients and 217 controls. Through principal component analysis (PCA) and hierarchical clustering for feature selection and SVM modeling for classification, the study demonstrated that tTG-DGP neoepitopes achieved exceptional diagnostic accuracy (99% sensitivity, 100% specificity), significantly outperforming traditional serologic tests (tTG-IgA/DGP-IgA), while also revealing a strong correlation between antibody-binding intensity and disease activity, with the highest levels in untreated CeD (32.5 ± 16.4 units) suggesting these neoepitopes could serve as novel biomarkers for both diagnosis and monitoring of mucosal healing [57].

Shemesh et al. [58] investigated the stratification of CD patients and healthy controls by analyzing naïve B-cell receptor repertoires. The study employed logistic regression with random forest for feature selection, focusing on CDR3 sequence clusters and biophysicochemical properties. The dataset included 92 samples (48 CeD patients and 44 controls) of naïve BCR heavy and light chains. Validation was performed using a 20% holdout approach with 1000 iterations. The model achieved an F1-score of 85% when combining data from both heavy and light chains. The research identified disease-associated BCR clusters with enriched V and J genes (such as IGHV1-18 and IGHJ3) and distinct 3-mer motifs, suggesting a possible genetic predisposition component in the development of CD through BCR-encoding genes [58].

In a study by dos Santos et al. [59], the goal was to develop an automated, observer-independent approach for classifying IgA endomysial antibody (EmA) test results for CD using ML, thereby addressing the subjectivity and labor intensity of manual evaluation. The researchers employed an SVM model with AdaBoost ensemble learning, utilizing a multiscale, rotation-invariant co-occurrence local binary pattern feature descriptor to analyze 2597 high-quality EmA test images classified into four categories: positive, negative, IgA deficient, and equivocal. The dataset was validated through expert evaluation and 10-fold cross-validation, with supplemental random under-sampling to address class imbalance. The primary model achieved a sensitivity of 82.84%, specificity of 99.40%, accuracy of 96.80%, and AUC values ranging from 99.61% to 100% across classes, with a mean processing time of 16.11 s per image. Limitations included class imbalance (e.g., only 0.5% IgA-deficient samples) and dependence on image quality [59].

The study by Carreras [60] aimed to predict and model CD using an autoimmune discovery gene panel and AI techniques, with a focus on identifying pathogenic genes such as BTLA. The research employed multiple ML and neural network models, including C5, logistic regression, Bayesian networks, discriminant analysis, KNN, LSVM, random trees, SVM, Tree-AS, XGBoost (linear and tree), CHAID, Quest, and C&R tree, as well as a multilayer perceptron neural network. The dataset consisted of gene expression profiles from 48 duodenal biopsies (26 CD patients and 22 controls) obtained from the publicly available GSE164883 dataset. Validation was performed through conventional bioinformatics, gene set enrichment analysis (GSEA), and immunohistochemical confirmation of BTLA protein expression in an independent series of 16 CD patients and 16 controls. The AI models achieved high prediction accuracy (95%–100%) in classifying CD, with key findings including the identification of immune checkpoint and immuno-oncology-related genes such as CASP3, CD86, CTLA4, FASLG, GZMB, IFNG, IL15RA, LAG3, and BTLA. The study also validated BTLA overexpression in CD at the protein level, linking it to inflammatory cells in the lamina propria [60].

Multiomics represents a comprehensive domain in biomedical research that covers a broad spectrum of biological levels, ranging from genomics to advanced layers such as proteomics and metabolomics, along with the intricate interplay between these levels. The fundamental feature of this expansive area of study is its intellectual intricacy, underscoring the importance of streamlining complexity to comprehend biological systems better. The development of advanced computational methods for managing multiomics data has opened up a new realm of research possibilities. These cutting-edge methods have not only fueled fundamental biological investigations but also hold promise for potential use in drug development and molecular systems engineering, particularly in complex conditions such as cancers. These advancements have paved the way for the realization of personalized medicine [71].

Numerous initiatives have been undertaken in multi-omics analysis concerning CD. Through an ongoing prospective study and multiomics analysis, researchers aimed to thoroughly investigate the impact of genetic and environmental risk factors on the longitudinal development of gut microbiota in infants who are at risk for developing CD. This includes examining potential triggers, such as gluten, before introducing solid foods. These analyses have revealed a range of microbial species, functional pathways, and metabolites, some of which have been linked to inflammation or immune system issues, while others are newly reported and may be specific to CD. This groundbreaking study provides unprecedented insights into significant taxonomic and functional shifts in the evolving gut microbiota of infants at risk of CD, establishing links between genetic and environmental risk factors and detrimental immunomodulatory and inflammatory effects through multiomics analysis [45]. Additionally, a longitudinal multiomics analysis of irritable bowel syndrome (IBS) diseases, encompassing CD patients, sought to explore the microbiome, metabolome, and epigenome of the subjects. The data identified a consistent elevation of the bacterial metabolite tryptamine in all IBS patients, stimulating colonic mucosal secretion and immune activation through inflammatory-related pathways [72, 73].

Classifying immune-related genes with different ML models has demonstrated the appropriate drug option for each group of celiac patients. Dysregulated immune-related genes, such as MR1, CCL25, and TNFSF13B, have been identified in CD using ML algorithms. Then, another artificial neural network (ANN) was designed to assess the diagnostic validity of these hub-immune-related genes. Comprehension of the exact pathway of celiac-related gene expression has led to the discovery of potential therapeutic options [20].

The integration of AI with multiomics data presents transformative potential that extends beyond improved diagnostics to fundamentally reshape treatment paradigms [74]. These technologies enable a precision medicine approach by identifying distinct patient subgroups based on molecular signatures, predicting individual treatment responses, and uncovering novel therapeutic targets [75]. For instance, ML analysis of genomic, proteomic, and metabolomic profiles could guide dietary modifications beyond gluten-free diets or identify candidates for emerging therapies like tight junction regulators or transglutaminase inhibitors [76].

For instance, a recent study applied ML to predict clinical outcomes in potential CD, advancing precision medicine approaches. In this study, four ML models were trained to predict the grade of villus atrophy based on serological and molecular markers. Anti-transglutaminase IgA, HLA-DQ2, and HLA-DQ8 were the primary positive markers. IL-2, IL-12, IL-21, and anti-endomysial antibodies were also used to train the ML models. Family history of thyroiditis and height of villi in the first biopsy were taken into account. The accuracy rate of all models was above 75% which indicates the high predictive potential of integrating multiomics and ML[77].

The multiomics analysis of CD offers a more comprehensive understanding of the pathways dysregulated in this condition. Therefore, the clinicians and pharmacologists could work on better therapeutic interventions than a gluten-free diet. Prolyl endopeptidases (PEP) are the enzymes that detoxify dietary gluten. Sphingomonas capsulate PEP (SC PEP) is one of the peptidases that was redesigned using ML and physical parameters in Xiao's study to be a more effective therapeutic option for CD patients. M-24 and EP-B2 combination with SC PEP is a new mutant of SC PEP designed by a ML model considering the environmental conditions of the digestive system and patients' HLA DQ2 or HLA DQ8 positive antigen-presenting cells to help CD patients [19]. More studies evaluating the redesign of enzymes and proteins using multi-omics and AI can yield promising results for treatment.

## 4. Conclusions

The integration of AI and multiomics data holds transformative potential for CD diagnosis and management. As these advancements continue to progress, their integration into personalized tools, early diagnosis, novel drug development, treatment customization, and long-term complication prediction, like osteoporosis and lymphoma, will likely shape a future where individuals with CD can lead healthier, more resilient lives. Our review highlights substantial advancements in both diagnostic accuracy and patient care, driven by innovative ML and deep learning techniques applied to multiomics profiles. Notably, AI-assisted methods, such as VCE analysis, have shown sensitivities exceeding 90%, facilitating more timely diagnoses while reducing the invasiveness associated with traditional biopsy procedures. Studies demonstrate that AI can significantly enhance the interpretation of complex datasets, leading to precise identification of CD and stratification of patients based on their response to gluten exposure. Furthermore, the emergence of novel biomarkers through multiomics analysis underscores a promising shift towards a more individualized approach to CD management. These biomarkers not only aid in diagnosis but also enable monitoring of disease progression and response to dietary interventions, ultimately improving patient outcomes.

While AI and multiomics hold great potential for improving CD management, significant challenges must be overcome before these approaches can be widely adopted in clinical practice. One major obstacle is the difficulty of integrating these technologies into routine healthcare workflows, as many medical centers lack the necessary infrastructure to handle large-scale genomic data or AI-based analysis tools. For clinicians to adopt these methods, the systems must be user-friendly and compatible with existing electronic health records. Cost is another critical factor, as healthcare institutions, especially those with limited resources, will need clear evidence that these technologies provide sufficient benefits to justify their expense. Data privacy and security remain pressing concerns, particularly when handling sensitive genetic and health information, which requires strict adherence to regulations such as the General Data Protection Regulation (GDPR) and the Health Insurance Portability and Accountability Act (HIPAA).

Additionally, regulatory frameworks for AI-driven diagnostics are still evolving, and more straightforward guidelines from agencies such as the Food and Drug Administration (FDA) and the European Medicines Agency (EMA) will be necessary to ensure these tools meet clinical standards. Standardization is another key challenge, as differences in data collection, analytical methods, and interpretation across platforms may lead to inconsistent results between institutions. To address these barriers, collaboration among researchers, clinicians, policymakers, and industry will be essential to establish reliable protocols, demonstrate real-world effectiveness, and ensure these innovations can be confidently integrated into patient care.

## Figures and Tables

**Table 1:** AI and multiomics studies in celiac disease.

Study (year)	Objective	AI model	Dataset size (samples)	Validation method	Performance metrics	Reported limitations	Key findings	References
Ciaccio et al. (2010)	Classify video capsule endoscopy images (celiac vs. controls) using texture/brightness features	Threshold classifier, incremental learning classifier	11 celiacs, 10 controls (200 images/videoclip)	Exemplar/test set split (6:5 train, 5:5 test)	Sensitivity: 80%–88%, specificity: 80%–96%	Small sample size; excluded clips with >10% fluid/bubbles; manual feature selection	Celiac images showed linear texture trends along the intestine; high classification accuracy	[49]
Wang et al. (2020)	Develop a deep-learning module to diagnose celiac disease from video capsule endoscopy (VEC) images	ResNet50, inception-v3 with recalibration	37 participants (21 CD, 16 healthy)	10-fold cross-validation	Accuracy: 95.94%, sensitivity: 97.20%	Small dataset. Patient-wise results not statistically significant. Longer computation time	High performance with recalibration module	[50]
Zammit et al. (2023)	Quantify CD severity using MLA vs. expert VCE scoring	ResNet50-based MLA	334,080 frames (35 CD)	Krippendorff's α, *R*^2^	*α* = 0.935 (expert-MLA agreement), *R*^2^ = 0.906 (whole SI)	Single-center bias; no external validation	MLA replicated expert scoring; enabled quantitative CD severity assessment	[51]
Stoleru et al. (2022)	Develop a noninvasive, efficient ML model for celiac disease detection	Sobel filters, custom kernels, KNN, SVM	109 videos (45 healthy, 65 celiac)	51 videos, used for the training data set 51 Videos, used for the test set 7 Videos, kept for real-time testing	Accuracy: 94.1%; F1-score: 94%	Limited dataset, potential overfitting, lack of validation for other diseases	Linear SVM outperformed KNN; achieved 94.1% accuracy with simple filters	[52]
Wei et al. (2019)	Classify CD from duodenal biopsies using deep learning.	ResNet-50	1230 slides (1048 patients)	Independent test set (212 slides)	Accuracy: 95.3%, AUC: 0.993 (CD)	Single-center bias; limited nonspecific duodenitis samples	High performance for CD; CAM visualizations validate pathological relevance	[53]
Koh et al. (2021)	Automate CD detection from biopsy images	RF, SVM, KNN, AdaBoost, bagged trees, discriminant subspace	120 (82 CD, 38 controls)	10-fold cross validation	88.89% accuracy (H&E), 82.92% (RGB), 72% (multiclass)	Small dataset, image variability, biopsy orientation	Automated biopsy analysis reduces pathologist workload and variability	[54]
Syed et al. (2019)	Differentiate environmental enteropathy (EE), CD, and healthy duodenal biopsies	Custom CNN + DNN	102 patients (3118 images)	10-fold cross-validation	93.4% case accuracy; 2.4% false-negative rate	Staining heterogeneity, small EE cohort, broad control criteria	CNN identified secretory cells as key discriminative features	[55]
Gruver et al. (2023)	Quantify celiac disease features (IELs, villus blunting, and crypt hyperplasia)	DenseNet2 CNN	116 (10 training)	Train-test split (10 training, 106 test); correlation with pathologist Marsh scores	96.4% alignment in diet-response biopsies; strong correlation for IELs/villus blunting (*p* < 0.0001)	Small sample size, class imbalance, crypt hyperplasia weakly correlated, no external validation	ML classifiers reduced subjectivity and captured continuous data beyond ordinal Marsh scores	[56]
Choung et al. (2019)	Identify tTG-DGP neoepitopes for CD diagnosis/monitoring	Random forest, SVM (scikit-learn)	Discovery: 90 CD, 79 controls; validation: 82 CD, 217 controls	PCA, SVM hyperplane optimization, and ROC analysis	Sensitivity: 99%, specificity: 100% (diagnosis); 84%/95% (mucosal healing)	Synthetic peptides, no 3D structure validation, IgA deficiency gap	tTG-DGP neoepitopes outperform tTG-IgA/DGP-IgA tests for diagnosis and monitoring mucosal healing	[57]
Shemesh et al. (2021)	Stratify CD using naïve BCR repertoires	LR with RF feature selection, MIL for motifs	92 (48 CD, 44 controls)	20% holdout cross-validation (1K iterations)	F1-score: 85% (HC + LC)	Potential non-naïve cell contamination; no external validation	Identified disease-linked BCR clusters with genetic signatures (e.g., IGHV1-18)	[58]
Santos et al. (2019)	Automate IgA endomysial antibody (EmA) test classification for celiac disease using ML	SVM with AdaBoost, multiscale LBP feature descriptor	2597 (274 positive, 2260 negative, 13 IgA deficient, and 50 equivocal)	Expert evaluation, 10-fold cross-validation, random under-sampling (supplemental model)	Sensitivity: 82.84%; specificity: 99.40%; accuracy: 96.80%; AUC: 99.61%–100%	Class imbalance (e.g., 0.5% IgA-deficient samples); image quality dependency	First ML-based automated EmA classifier; high specificity and rapid processing (16.11 s/image)	[59]
Carreras (2022)	Predict and model celiac disease using an autoimmune gene panel and AI	C5, logistic regression, Bayesian network, discriminant analysis, KNN, LSVM, SVM, XGBoost, CHAID, Quest, C&R tree, neural network (multilayer perceptron)	48 (26 celiac, 22 controls)	GEO2R, GSEA, immunohistochemistry (independent validation)	95%–100% accuracy	Small sample size; requires larger validation	Identified immune-related genes (BTLA, CASP3, LAG3, etc.); validated BTLA overexpression in celiac disease	[60]
DiPalma et al. (2021)	To improve computational efficiency of histology image classification using knowledge distillation	ResNet (teacher and student models)	Celiac Disease (CD): 1364 patients	Bootstrapping (10,000 iterations)	Accuracy: 87.06%, F1-Score: 75.44% (for CD)	Requires high-resolution WSIs during training; potential biases in staining/slide preparation	Knowledge distillation with self-supervision improves performance at lower resolutions, reducing computational costs by up to 64x.	[61]
Zhou et al. (2017)	To develop a deep learning method for quantitative analysis of CD using video capsule endoscopy (VCE)	GoogLeNet (deep convolutional neural network)	11 celiac patients + 10 controls; approximately 4 clips per subject; 200 frames per clip	7-fold cross-validation; separate testing on 5 celiac + 5 control patients	Sensitivity: 100%, specificity: 100% (testing set and cross-validation); EC values significantly differentiated veroups (*p*=0.005)	Small dataset size; requires further validation with larger, blinded studies; unable to replace biopsy for definitive diagnosis yet	Deep learning method accurately distinguished celiac from control subjects; evaluation confidence (EC) correlated with disease severity (Marsh IIIA–IIIC); promising for automated, noninvasive celiac disease detection	[62]
Hujoei et al. (2018)	Identify undiagnosed celiac disease using ML	Logistic regression, random forest, SVM, neural networks	816 (408 cases, 408 controls)	10-fold cross-validation	AUC: 0.49–0.53	Poor discriminatory power, reliance on symptomatology, incomplete family history data	Case-finding based on symptoms was ineffective; models performed no better than chance	[63]
Dreyfuss et al. (2024)	To develop ML models for early identification of celiac disease autoimmunity	XGBoost, logistic regression, random forest, multilayer perceptron, decision tree	Train: 176,970 (677 cases, 176,293 controls); test: 41,240 (153 cases, 41,087 controls)	Distinct test set evaluation	AUC: XGBoost (0.86), logistic regression (0.85), random forest (0.83), MLP (0.80), decision tree (0.77)	Lack of unstructured data (e.g., clinical notes), potential undiagnosed controls, heterogeneity not captured	XGBoost performed best; model uses common lab results and demographics to identify at-risk patients	[64]
Shen et al. (2023)	To identify immune-related biomarkers and develop diagnostic models for celiac disease	ANN (artificial neural network)	132 (110 CD, 22 controls)	Independent validation cohort (GSE164883)	Training set AUC: 0.793, test set AUC: 0.821	Small cohort, limited clinical/genetic data, lack of functional validation	MRI, CCL25, and TNFSF13B are key biomarkers; ANN showed strong diagnostic potential; Tetradioxin identified as a potential therapeutic drug	[20]
Vicnesh et al. (2019)	Develop computer-aided detection (CAD) for celiac from capsule images	DAISY descriptors, Shannon entropy, PSO	52 CD videos, 55 healthy (2,140 images)	10-fold cross-validation	Accuracy: 89.82%, sensitivity: 94.35%	Limited to video capsule endoscopy	Demonstrated feasibility of automated detection	[65]
Molder et al. (2023)	Automated detection of endoscopic markers	Layered CNN	505 patients (182 CD, 323 controls)	Train–validation split	Sensitivity: 99.67%, positive predictive value (PPV): 98.07%	Patchy VA may bias image selection. No standardization of angle/proximity	Exceptional sensitivity but potential overfitting	[66]
Saken et al. (2021)	Enhance CD diagnosis in endoscopy	Hybrid ML (DWT, multilevel thresholding)	353 patients (1661 images)	10-fold cross-validation	Accuracy: 94.79%, sensitivity: 94.29%	Small private dataset (11 CD/76 controls). Class imbalance (bias toward early stages). Image quality issues (reflections, bubbles, blurring)	Robust performance with hybrid approach	[67]
Jaeckle et al. (2025)	To develop a machine learning model for diagnosing celiac disease from duodenal biopsy images	Multiple-instance learning (weakly supervised)	3383 WSIs (training, 4 hospitals), 644 WSIs (independent test set)	Independent test set from a fifth hospital; compared to 4 pathologists	Accuracy, sensitivity, specificity >95%; AUC >99%	Requires digitized biopsies; generalizability to nonbiopsy-based diagnosis not tested; regulatory approval needed	Achieved pathologist-level performance; strong generalizability; potential to revolutionize biopsy-based diagnosis	[68]

## Data Availability

The data sharing is not applicable to this article as no new data were created or analyzed in this study.

## Nomenclature

AI: Artificial intelligence
ANN: Artificial neural network
BTLA: B and T lymphocyte-associated
CD: Celiac disease
CDSS: Clinical decision-support system
CE: Capsule endoscopy
CNN: Convolutional neural network
CT: Computed tomography
DGP: Deamidated gliadin peptides
EmA: Endomysial autoantibody
GRDs: Gluten-related diseases
GSEA: Gene set enrichment analysis
H&E: Hematoxylin and eosin
IBS: Irritable bowel syndrome
IEL: Intraepithelial lymphocyte
ML: Machine learning
MRI: Magnetic resonance imaging
NCGS: Non-celiac gluten sensitivity
PEP: Prolyl endopeptidases
PET: Positron emission tomography
RGB: Red-green-blue
SC PEP: Sphingomonas capsulate PEP
tTG: Tissue transglutaminase.
